# Parallel Changes in Positive Youth Development and Self-awareness: the Role of Emotional Self-regulation, Self-esteem, and Self-reflection

**DOI:** 10.1007/s11121-022-01345-9

**Published:** 2022-01-28

**Authors:** Esther C. A. Mertens, Maja Deković, Monique van Londen, Ellen Reitz

**Affiliations:** Department of Clinical Child and Family Studies, Utrecht University, Heidelberglaan 1, 3584 CS Utrecht, The Netherlands

**Keywords:** Self-awareness, Emotional self-regulation, Self-esteem, Self-reflection, Parallel change, School-based intervention, Positive youth development

## Abstract

The aim of the present study was to examine whether early adolescents’ positive development (i.e., resilience and psychological wellbeing) could be enhanced by stimulating three dimensions of self-awareness: emotional self-regulation, self-esteem, and self-reflection. An experimental field study (randomized controlled trial) was conducted. Seventh grade students (*N* = 1299; *M*_age_ = 12.38; 54% boys) completed multiple assessments at the beginning, during, and immediately after the intervention or at parallel time points for students in the control condition. Changes in the assessed constructs were examined with parallel process latent growth curve models. The results showed that increases in emotional self-regulation and self-esteem were related to concurrent increases in both resilience and psychological wellbeing. Changes in self-reflection were, however, not related to changes in these outcomes. The trajectories of change and parallel processes were similar in the intervention and control condition. These findings suggest that interventions aiming to stimulate positive youth development might be optimized by also focusing on youth’s self-awareness, though more knowledge about how self-awareness can be stimulated best is needed. This trial was registered in the Dutch Trial Register, number NL6371 (old number: NTR6554), on July 3, 2017.

**Supplementary Information:**

The online version contains supplementary material available at 10.1007/s11121-022-01345-9.

## Introduction

Studying how positive youth development can be stimulated is pivotal since positive outcomes can provide foundations for healthy development as well as function as protective factors for decreasing problem behaviors (Greenberg et al., 2017). Especially youth’s resilience (i.e., the ability to adapt to change and stressful events in a healthy and flexible way; Catalano et al., 2004) and psychological wellbeing (i.e., the presence of positive feelings and absence of negative feelings; Ravens-Sieberer & The European KIDSCREEN Group, 2006) appear important positive outcomes to strengthen (Benson et al., 2007; Catalano et al., 2004). Self-awareness has been indicated by the Collaborative for Academic, Social, and Emotional Learning (CASEL) as an important competence that provides a foundation for enhanced positive youth development. Therefore, the aim of the present study is to examine parallel changes in self-awareness and both youth’s resilience and psychological wellbeing. Unraveling associations between changes in one variable and changes in another variable provides not only insights in one’s development, but can also contribute to understanding individual differences (Cheong et al., 2003). This knowledge can subsequently inform theory and optimization of interventions (O’Rourke & MacKinnon, 2018).

### Self-awareness and Positive Development

Self-awareness is a multidimensional overarching theoretical concept that can be defined as “understanding your own emotions, values, and personal goals” (Greenberg et al., 2017, p. 14). It concerns the tendency to focus and reflect on one’s inner processes and own experiences, as well as being aware of other’s perceptions (Sutton, 2016). A high level of self-awareness is characterized by recognition of one’s own thoughts, feelings, and actions, as well as feeling accepted by others (Greenberg et al., 2017; Sutton, 2016). Based on this conceptualization of self-awareness, the competencies emotional self-regulation, self-esteem, and self-reflection seem to be key dimensions of self-awareness (Sutton, 2016).

According to CASEL’s conceptual framework (Greenberg et al., 2017), improving self-awareness can, in turn, improve one’s positive development. This proposed link is supported by empirical research. For instance, Morrish et al. (2018) concluded in their review that research has linked adaptive emotional self-regulation to more resilience and better psychological wellbeing. Also, higher self-esteem has been associated with better adjustment, such as mental health and happiness, and can buffer the impact of stressful events (see for a review Mann et al., 2004). Research relating self-reflection to positive development is, however, inconclusive. For instance, Elliot and Coker (2008) found in a community sample that more self-reflection was related to more happiness, whereas, in a comparable community sample, Lyke (2009) did not find a relation between self-reflection and happiness nor between self-reflection and life satisfaction. Thus, all three key dimensions of self-awareness have been linked to positive youth development, though the exact role of self-reflection remains somewhat ambiguous.

### An Intervention Context

An intervention that aims to enhance youth’s positive development by stimulating the three key dimensions of self-awareness, i.e., their emotional self-regulation, self-esteem, and self-reflection, is Rock and Water (R&W; Ykema, 2002, 2018). R&W aims to improve students’ emotional self-regulation, self-esteem, and self-reflection by using a combination of a physical and psychological approach (i.e., a psychophysical approach). For instance, the intervention theorizes that emotional self-regulation is stimulated by making students aware of physical representations of their emotions, such as muscle tension and high breathing. Students are taught how to actively relax their muscles and lower their breathing to become calm. Self-esteem is addressed explicitly by encouraging an upright body posture and implicitly by creating experiences of success during the lessons. Self-reflection is stimulated by a moment of reflection after each exercise guided by questions of the R&W trainer (Ykema, 2002, 2018).

The intervention is based on the theory of the “R&W house” (Ykema, 2002, 2018). The competencies emotional self-regulation, self-esteem, and self-reflection are the pillars of the house. Developing these basic competencies is theorized to enable students to develop themselves on broader competencies such as resilience and psychological wellbeing. Small-scaled evaluation studies using single group pretest–posttest design showed improvements in participants’ resilience, positive identity, and coping styles (Ykema et al., 2006). Additionally, a recent large-scaled randomized controlled trial (RCT) showed improvements in participants’ coping strategies, self-regulation, and self-efficacy (De Graaf et al., 2016). However, the role of the three dimensions of self-awareness, the pillars of the R&W house, in these intervention effects remains unclear.

### The Current Study

In the present study, we empirically tested the CASEL’s framework by examining whether changes in self-awareness (i.e., emotional self-regulation, self-esteem, and self-reflection) were related to concurrent changes in youth’s positive development (i.e., resilience and psychological wellbeing). In addition, we examined in an experimental field study whether these parallel changes could be affected by an intervention, R&W (Ykema, 2002, 2018). Experimental manipulation empowers the argument that changes in one construct could be responsible for changes in the other construct (Kazdin, 2007). The intervention was implemented for 7th grade students following the prevocational education track (i.e., the lowest level of three educational tracks in the Dutch secondary school system). In general, this group of students has an increased risk of developing behavioral and peer related problems (Stevens & De Looze, 2018), making it important to gain more insight in the way their positive development is shaped. We hypothesized that changes in adolescents’ emotional self-regulation, self-esteem, and self-reflection would be related to concurrent changes in their resilience and psychological wellbeing. In addition, we hypothesized that adolescents in the R&W condition would show a stronger increase in emotional self-regulation, self-esteem, and self-reflection which would be related to a stronger concurrent increase in resilience and psychological wellbeing, compared to adolescents receiving care as usual (i.e., control condition).

Our study adds to the literature in four ways. First, even though self-awareness is a multidimensional concept (Greenberg et al., 2017), often only one dimension of self-awareness is assessed (e.g., Weytens et al., 2014). It is important to examine multiple dimensions of self-awareness as these might relate differently to positive youth development. Second, despite the focus of CASEL’s conceptual framework on youth’s positive development, studies predominantly focus on maladjustment and problem behaviors (e.g., Blossom et al., 2020), rather than on adjustment and positive outcomes, especially when youth have an increased risk of developing problems. In order to gain insights in how the positive development of these at risk youth can be stimulated, it is eminent to also focus on positive outcomes (Benson et al., 2007). Third, although metacognitions such as self-awareness develop particularly during early adolescence, as adolescents’ cognitive and emotional capacities increase (Barber, 2005), only few studies explicitly focus on (early) adolescents. Instead, studies focus on children (e.g., Rimm-Kaufman & Hulleman, 2014), both children and adolescents (e.g., Morrish et al., 2018), or older youth (e.g., Bakker & Rickard, 2018). Fourth, previous longitudinal research has examined (dimensions of) self-awareness as predictor of later positive outcomes. However, the developmental trajectories of self-awareness and positive outcomes may be related. By examining parallel change, potential mechanisms that shape development may be uncovered (Cheong et al., 2003).

## Method

### Design and Procedure

Data for the current study were collected as part of a larger project examining the effectiveness of R&W by means of a 2-year RCT (see Mertens et al., 2018 for the study protocol). This RCT consisted of three intervention conditions that differed in the number of people involved in the intervention (i.e., light: a core team of teachers; standard: all teachers; plus: all teachers and parents) and a control condition (i.e., care as usual).

**Fig. 1:**
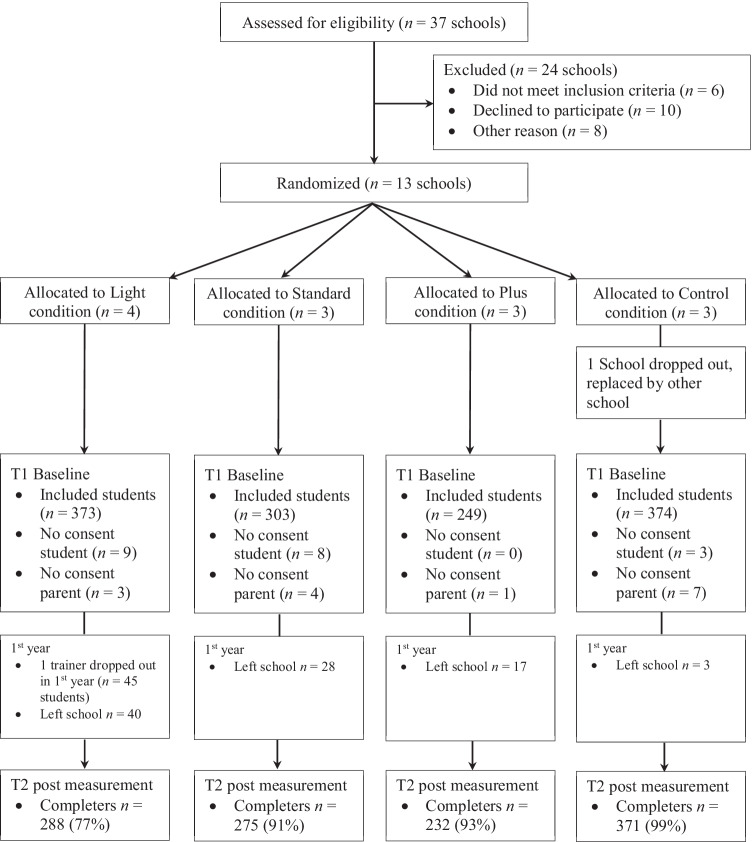
Flow chart

Schools were eligible for inclusion if they offered the prevocational education track and had not implemented R&W in the last 2 years. Using an online random number generator, the first author randomly allocated 13 schools in urban and rural areas throughout the Netherlands to the conditions (1:1:1:1). To enhance an equal distribution of students over the conditions, schools were stratified by school size (small to moderate sized schools with < 100 students in the 7th grade, large schools with > 100 students in the 7th grade) in blocks of four (i.e., the number of conditions). After randomization but before the start of data collection, one school in the control condition dropped out due to a change in school management and was replaced by another school (see Fig. 1). As the focus in the present study is on parallel change rather than its effectiveness, we merged the data of the three intervention conditions to form one intervention condition. Additionally, we only used data of the first year of the RCT as intervention effects were mostly established during the first part of intervention (see Mertens et al., 2021).

Students completed questionnaires before the intervention started, at baseline (T0; October/November 2017), after each third intervention lesson of the 14 lessons (4 measurement points), and after completing the first year of the intervention (T5; March/April 2018). The interim measurements were shortened questionnaires based on face validity and factor loadings of the items in previous studies. Students gave active informed consent for completing the questionnaires, and parents gave passive informed consent for the participation of their child. This trial was approved by the Ethical Committee of the Faculty of Social and Behavioral Sciences of Utrecht University (FETC17-05) and registered in the Dutch Trial Registration, number NL6371 (old number NTR6554; see for protocol Mertens et al., 2018).

### Participants

At baseline, 1299 7th grade students with an average age of 12.38 years (*SD* = .62) participated. About half of the participants (*n* = 661; 54%) were boys and most (*n* = 815; 69%) had a Western background.^1^ In the R&W condition, students (*n* = 925) were on average 12.35 years old (*SD* = .61) of whom 462 (52%) were boys and 617 (73%) had a Western background. In the control condition, students (*n* = 374) were on average 12.47 years old (*SD* = .64) of whom 199 (57%) were boys and 198 (59%) had a Western background.

Students in the R&W condition were slightly younger (*F*(1,1230) = 10.46, *p* = .001, *η*^2^_partial_ = .008), and more had a Western background (*χ*^2^(1) = 20.07, *p* < .001, *φ* =  −.130) than students in the control condition. Age and ethnic background were therefore added as a covariate in the analyses.

### Attrition

In total, 472 (36%) students completed the questionnaires at all waves and 1170 (90%) completed the questionnaires at least 3 of the 6 waves. Chi-square difference tests and a MANOVA showed that students without missing data did not differ from students with missing data on gender (*χ*^2^(1) = 1.30, *p* = .254, *φ* = .032), age, and outcomes (*F*(6,1210) = 1.57, *p* = .152, *η*^2^_partial_ = .008). Concerning ethnic background, students without missing data had slightly more often a Western background than students with missing data (*χ*^2^(1) = 7.61, *p* = .006, *φ* = .080).

### Conditions

#### R&W

Students received 14 R&W lessons of the manualized program (Ykema, 2002, 2018). The lessons were implemented weekly during 90-min physical education lessons. Trainers were teachers of the schools, mostly physical education teachers, who completed the 3-day training to become certified R&W trainers. During the lessons, students participated in physical games and exercises, reflected on the exercises, shared and discussed their thoughts with each other, and addressed how to use the learned skills in their daily lives.

Communication during the intervention is framed within the symbolic principles of “rock” (i.e., an uncompromising attitude in which one is able to resist pressure from others) and “water” (i.e., a flexible and cooperative attitude in which one is open to opinions, thoughts, and feelings of others) representing opposite ends of a continuum (see the study protocol for more information Mertens et al., 2018).

Treatment fidelity was assessed with self-reports of the R&W trainers and with observations of 67 R&W lessons by 3 R&W experts. Overall, the majority of the R&W lessons were indeed implemented and fidelity to the manual was moderate to high (see Mertens et al., 2021 for an elaborate description of treatment fidelity).

#### Control

Schools in the control condition implemented care as usual which differed between schools. For instance, students were assigned a teacher as personal coach to discuss their wellbeing. Additionally, schools facilitated actions to prevent or stop bullying such as a project week about “being different,” an anti-bullying contract, and discussing bullying in the class or issues were addressed by an “anti-bulling coordinator.”

### Measurements

#### Positive Outcomes

##### Resilience

The ability to adapt to change and stressful events in a healthy and flexible way was assessed with 3 items (e.g., “Able to adapt to change.”) of the Connor-Davidson Resilience Scale — short version (Davidson & Connor, 2017) rated on a 5-point Likert-type scale (0 = *not true at all* to 4 = *true nearly all the time*; Cronbach’s *α* = .56–.70).

##### Psychological Wellbeing

The presence of positive feelings and absence of negative feelings were measured using the subscale Psychological wellbeing of the KIDSCREEN-27 (Ravens-Sieberer & The European KIDSCREEN Group, 2006) with 2 items (e.g., “Past week, have you been in a good mood?”) rated on a 5-point Likert-type scale (1 = *never* to 5 = *always*; Cronbach’s *α* = .67–.81).

#### Self-awareness

##### Emotional Self-regulation

The ability to control emotions and access to emotion regulation strategies was measured using 4 items (e.g., “When I’m upset, I become out of control.”) of the Difficulties in Emotion Regulation Scale (Anderson et al., 2016) answered on a 5-point Likert-type scale (1 = *almost never* to 5 = *almost always*). All items were recoded so that high scores indicate high levels of emotional self-regulation (Cronbach’s *α* = .73–.84).

##### Self-esteem

Feelings of global self-worth was assessed with 3 items (e.g., “I am satisfied with myself.”) of the subscale global self-perception of the Self-perception Profile (Harter, 1988) answered on a 4-point Likert-type scale (1 = *completely not true* to 4 = *completely true*). Some items were recoded so that high scores indicated high levels of self-esteem (Cronbach’s *α* = .64–.71).

##### Self-reflection

Students completed 3 items (e.g., “I often think about how I feel about something.”) of the subscale Engage in reflection of the Self-reflection and Insight Scale (Sauter et al., 2010) to measure the extent to which students inspect and evaluate personal thoughts, feelings, and behaviors. The items were preceded by a definition of self-reflection and answered on a 6-point Likert scale (1 = *disagree strongly* to 6 = *agree strongly*; Cronbach’s *α* = .74–86).

### Statistical Analyses

An intention-to-treat approach was employed, that is, all students who were randomized were included in the analyses regardless of their level of attendance to the assigned condition. In order to include all participants in the models, full information maximum likelihood (FIML) procedures were used with robust maximum likelihood estimation (MLR) to obtain parameter estimates, which is robust to non-normality and non-independence of observations. Clustering at school level was taken into account using the complex sample cluster feature of M*plus* (version 8.2; Muthén & Muthén, 2017). This conservative clustering procedure provides unbiased estimates of the standard errors (Muthén & Muthén, 2017). Clustering at class level was not taken into account due to generally low and nonsignificant intraclass correlations at this level (e.g., Cross et al., 2016).

Parallel changes were analyzed by testing a series of parallel process latent growth curve models (LGC; Cheong et al., 2003). First, we modeled unconditional LGC models for each outcome to assess change over time (regardless of condition). LGC models estimate two latent variables that describe the average initial level (i.e., intercept) and average change over time (i.e., slope) based on growth curves of individual participants (Muthén & Muthén, 2017). Model fit was determined based on the RMSEA (good fit < .05), CFI (good fit > .95), and SRMR (good fit < .08; Schermelleh-Engel et al., 2003). Second, the LGC model of one dimension of self-awareness was combined with the LGC model of one positive outcome into one parallel process growth curve model by regressing the slope of the positive outcome on the slope of the dimension of self-awareness. Students’ age and ethnic background were added as covariates. A significant regression coefficient between the two slopes indicated that the trajectories of change of the constructs were related. Third, a dummy variable representing the intervention condition (1 = intervention, 0 = control) was added to the model by regressing the slopes of the positive outcome and of the dimension of self-awareness on the dummy variable. To control for initial differences between conditions, we also regressed the intercepts of the positive outcome and of the dimension of self-awareness on the dummy variable. Whether the intervention affected the parallel change of the constructs was examined by modeling the indirect path from condition to outcome via the dimension of self-awareness.

## Results

### Unconditional Growth Models

The descriptives of the concepts per measurement point per condition are shown in Table 1. To assess change over time, we modelled unconditional linear growth models for each concept separately. Factor loadings were set at 0, 2, 3.5, 5, 6.5, and 7.5, representing a time span between the measurement points of respectively 4 weeks, 3 weeks, 3 weeks, 3 weeks, and 2 weeks. All models showed an acceptable to good model fit indicating that the linear growth models adequately described the changes in the concepts. Across all participants, there was a slight increase in resilience, psychological wellbeing, emotional self-regulation (*p* = .051), and self-esteem. Regarding self-reflection, there was a slight decrease over time (see Table 2 for unstandardized estimates of the intercepts and slopes and the model fit statistics of the linear growth models).

**Table 1:** Descriptives of the concepts per measurement point for each condition

	R&W						Control					
	T0 (baseline)	T1	T2	T3	T4	T5 (post)	T0 (baseline)	T1	T2	T3	T4	T5 (post)
	*M* (*SD*)	*M* (*SD*)	*M* (*SD*)	*M* (*SD*)	*M* (*SD*)	*M* (*SD*)	*M* (*SD*)	*M* (*SD*)	*M* (*SD*)	*M* (*SD*)	*M* (*SD*)	*M* (*SD*)
Resilience	2.63 (.78)	2.90 (.80)	2.81 (.82)	2.88 (.82)	2.92 (.79)	2.77 (.78)	2.77 (.76)	2.69 (.87)	2.72 (.82)	2.76 (.82)	2.84 (.74)	2.90 (.80)
Psychological wellbeing	4.07 (.70)	4.05 (.79)	4.08 (.79)	4.03 (.83)	4.12 (.78)	4.11 (.77)	4.11 (.71)	4.21 (.67)	4.20 (.76)	4.16 (.74)	4.17 (.78)	4.14 (.77)
Emotional self-regulation	3.99 (.86)	3.79 (.92)	3.82 (.94)	3.76 (.96)	3.87 (1.00)	4.09 (.94)	4.07 (.84)	4.07 (.84)	4.15 (.86)	4.09 (.88)	4.07 (.91)	4.13 (.89)
Self-esteem	3.26 (.64)	3.31 (.65)	3.31 (.65)	3.29 (.66)	3.34 (.63)	3.38 (.64)	3.33 (.62)	3.38 (.64)	3.43 (.62)	3.37 (.67)	3.42 (.64)	3.34 (.67)
Self-reflection	2.95 (1.24)	2.98 (1.29)	2.90 (1.29)	2.97 (1.33)	2.94 (1.40)	2.86 (1.32)	3.09 (1.21)	3.16 (1.32)	2.91 (1.32)	2.99 (1.27)	2.94 (1.40)	2.95 (1.44)

**Table 2:** Unstandardized estimates of intercepts and slopes and model fit statistics of unconditional linear growth models

	Parameters		Model fit		
	Estimate (*SE*)	*p*	RSMEA	CFI	SRMR
Resilience			.037	.98	.051
Mean intercept	2.717 (.052)	<.001			
Variance intercept	.235 (.031)	<.001			
Mean slope	.019 (.005)	<.001			
Variance slope^a^	.001 (.001)	.233			
Psychological wellbeing			.035	.97	.076
Mean intercept	4.082 (.043)	<.001			
Variance intercept	.247 (.022)	<.001			
Mean slope	.004 (.002)	.038			
Variance slope	.003 (.001)	.002			
Emotional self-regulation^b^			.059	.95	.083
Mean intercept	3.928 (.054)	<.001			
Variance intercept	.345 (.044)	<.001			
Mean slope	.009 (.004)	.051			
Variance slope^1^	.002 (.001)	.060			
Self-esteem			.013	1.00	.024
Mean intercept	3.283 (.030)	<.001			
Variance intercept	.262 (.018)	<.001			
Mean slope	.011 (.002)	<.001			
Variance slope	.002 (.000)	<.001			
Self-reflection			.050	.96	.066
Mean intercept	3.014 (.058)	<.001			
Variance intercept	.660 (.065)	<.001			
Mean slope	−.015 (.006)	.016			
Variance slope	.007 (.001)	<.001			

Standardizing estimates is complicated in multilevel models as variances used for standardization are located at the within and between levels (Muthén & Muthén, 2017). Since it is not our aim to compare multiple predictors within a model, standardized estimates are not necessary. Therefore, we reported the unstandardized variances

^a^Although the slope variance is not significant (or only marginally significant), these variables were included in the analyses, because power to detect slope variance is low due to a too conservative standard chi-square test — the true distribution of variances in LGC models follows a different distribution (Stoel et al., 2006)

^b^A quadratic LGC model did not significantly improve model fit

### Parallel Change

All parallel process LGC models showed a good model fit. Increases in emotional self-regulation were related to increases in both resilience and psychological wellbeing. Also, increases in self-esteem were related to increases in resilience as well as psychological wellbeing. Changes in self-reflection, however, were not related to changes in resilience nor to changes in psychological wellbeing (see Table 3).

**Table 3:** Unstandardized parameter estimates and model fit of the parallel process latent growth models

	Path b		Model fit		
	*B* (*SE*)	*p*	RMSEA	CFI	SRMR
*Resilience*					
Emotional self-regulation	.38 (.17)	.022	.037	.95	.047
Self-esteem	.94 (.08)	<.001	.023	.99	.032
Self-reflection	−.00 (.09)	.984	.030	.97	.044
*Psychological wellbeing*					
Emotional self-regulation	.52 (.14)	<.001	.035	.96	.052
Self-esteem	.91 (.15)	<.001	.026	.98	.045
Self-reflection	.04 (.08)	.612	.030	.96	.052

Path b: self-awareness → positive outcome

### Parallel Change in Intervention Context

When the intervention context was taken into account, the parallel changes between the dimensions of self-awareness and the positive outcomes remained the same and were similar in both the intervention condition and the control condition (i.e., care as usual); condition did not predict changes in the three dimensions of self-awareness nor in the two positive outcomes. Additionally, the indirect effects of condition on the positive outcomes through the dimensions of self-awareness were, unsurprisingly, not significant in all models (see Table 4).

**Table 4:** Unstandardized parameter estimates of the parallel process latent growth models in intervention context

	Path c		Path a		Path b		Indirect effect	
	*B* (*SE*)	*p*	*B* (*SE*)	*p*	*B* (*SE*)	*p*	*B* (*SE*)	*p*
*Resilience*								
Emotional self-regulation	.00 (.01)	.839	.00 (.01)	.821	.37 (.17)	.031	.00 (.00)	.816
Self-esteem	−.01 (.01)	.161	.01 (.01)	.200	.93 (.08)	<.001	.01 (.01)	.210
Self-reflection	−.00 (.01)	.615	.01 (.01)	.111	−.00 (.08)	.988	.00 (.00)	.988
*Psychological wellbeing*								
Emotional self-regulation	.00 (.01)	.838	−.00 (.01)	.834	.52 (.15)	<.001	−.00 (.00)	.838
Self-esteem	−.01 (.01)	.363	.01 (.01)	.481	.91 (.16)	<.001	.01 (.01)	.514
Self-reflection	.00 (.00)	.538	.02 (.01)	.090	.04 (.08)	.616	.00 (.00)	.605

Path c: condition → positive outcome; path a: condition → dimension self-awareness; path b: dimension self-awareness → positive outcome

## Discussion

In the current study, we examined whether changes in youth’s self-awareness were related to concurrent changes in positive development, as suggested in CASEL’s conceptual framework (Greenberg et al., 2017). We focused on multiple dimensions of self-awareness (i.e., emotional self-regulation, self-esteem, and self-reflection) and on positive development within an at risk group of early adolescents. In general, the adolescents showed a positive development by slightly improving on emotional self-regulation, self-esteem, resilience, and psychological wellbeing. Concerning self-reflection, however, youth slightly decreased. Increases in two dimensions of self-awareness, i.e., emotional self-regulation and self-esteem, were related to increases in resilience and psychological wellbeing, in line with previous research (e.g., Mann et al., 2004; Morrish et al., 2018). Changes in self-reflection were related neither to changes in resilience nor to changes in psychological wellbeing. These associations between the trajectories of self-awareness and of positive development were the same in an intervention context.

Concerning emotional self-regulation, stimulating youth’s competence in regulating their emotions might help them to up-regulate positive emotions and down-regulate negative emotions (Morrish et al., 2018). This ability enables youth to modify and appraise events in order to intensify positive affect or to buffer against negative affect and stressful events (Morrish et al., 2018; Quoidback et al., 2015). Experiencing positive emotions, and few negative emotions, may positively change situations (e.g., people smile at you), could facilitate an easy recollection of positive memories, and might shape the way an event is remembered (Quoidback et al., 2015). In sum, the positive affect together with decreases in negative affect can stimulate youth’s resilience and positive wellbeing.

Regarding self-esteem, high levels of self-esteem can reduce the impact of stressful events and failure (e.g., Blossom et al., 2020; Mann et al., 2004). Confident youth trust their abilities to effectively deal with daily problems (Blossom et al., 2020). They experience daily events as more positive and negative events as less important than youth with low levels of self-esteem, enabling them to reach their full potential and decreases their chances of developing internalizing or externalizing problem behaviors (Mann et al., 2004). In addition, when faced with failure, youth with high levels of self-esteem become extra motivated in pursuing their goals, while youth with low levels of self-esteem experience a further decline in self-esteem and positive affect, generalize the failure to other aspects of their selves, and disengage from their goals as they lose confidence in their abilities (Park et al., 2007). Thus, as for improvements in emotional self-regulation, increases in youth’s self-esteem can improve their psychological wellbeing and diminish the effects of negative events which may increase their resilience and ultimately stimulate their overall positive development.

In contrast, changes in self-reflection were not related to concurrent changes in youth’s positive development. Perhaps the mere act of reflecting on one’s own behavior, thoughts, and feelings is not enough for improvements in one’s development since this inspection can be independent of emotion and potential effects that arise due to this inspection (Stein & Grant, 2014). Also, other studies found no relations between self-reflection and youth’s positive development. However, they did find relations between self-insight and positive youth development (e.g., Lyke, 2009; Stein & Grant, 2014). Hence, rather than solely engage in self-reflection, it may be necessary to also *understand* one’s own behaviors, thoughts, and feelings. This understanding could contribute to one’s self-insight. Self-insight indicates the degree to which individuals understand their behavior, cognitions, and emotions — “ah-hah” moments of understanding (Stein & Grant, 2014). For instance, Lyke (2009) found that engaging in self-reflection was not related to happiness and life satisfaction, but high levels of self-insight were related to higher levels of these outcomes. Additionally, Stein and Grant (2014) found no direct relation between self-reflection and subjective wellbeing, but did find an indirect effect: More self-reflection was related to increases in self-insight which in turn could improve subjective wellbeing.The associations between the trajectories of change of dimensions of self-awareness and of youth’s positive development were similar in both the intervention and control group; in both conditions, students improved on emotional self-regulation, self-esteem, resilience, and psychological wellbeing. Although these improvements may represent adolescents’ typical development, this finding can also indicate that R&W and usual care at schools are equally effective which, in turn, suggests that care as usual in Dutch schools is of good quality. Notwithstanding this positive finding, it is important to examine how universal school-based interventions in general, and R&W specifically, can be optimized as intervention effects of this type of intervention are generally small (e.g., Mertens et al., 2020; Tanner-Smith et al., 2018). One way to optimize R&W may be by adjusting the dosage of some components. Interventions that address general interpersonal and emotional skills appear more effective when psychoeducation (3–6 exercises) and skill-building exercises (10–20 exercises) are included (De Mooij et al., 2020). During R&W, these components are implemented, but perhaps not in the right dosage. Thus, future research aiming to optimize interventions should focus on potentially effective components as well as the optimum dosage of these components.

Furthermore, our findings highlight positive developments in youth generally considered as “at risk.” Research examining adolescents following the prevocational education track often portrays these adolescents as “problematic students,” emphasizing the increased risk of maladjustment, such as behavioral problems, problems with peers, and substance use (e.g., Stevens & Vollebergh, 2018). However, based on the results of our study, this group of adolescents also shows positive developments on multiple competencies, i.e., emotional self-regulation, self-esteem, resilience, and psychological wellbeing, regardless of whether they received an intervention specially developed to target those outcomes or usual care of schools. In addition, the improvements in the competencies emotional self-regulation and self-esteem support the assumption that metacognitions such as self-awareness develop during early adolescence (Barber, 2005). Perhaps the current view of adolescents following the prevocational education track is more negative then needed due to the focus on their deficits, neglecting their potential strengths (Benson et al., 2007).

When evaluating the significance of the results, it is important to consider some strengths and limitations of the current study. Strengths of the study were the large sample of *early* adolescents considered as “at risk,” examining multiple dimensions of self-awareness, and multiple assessments of the concepts during the intervention. This enabled us to focus on the positive development of “at-risk” youth, to study a phase in which youth appear particularly sensitive for stimulation of their metacognitions, and to examine whether all dimensions of self-awareness have the same association with positive youth development. The multiple measurements enabled us to analyze parallel changes in self-awareness and positive development through latent growth models. A limitation of parallel process LGC models is, however, that the trajectories of change in the concepts are a function of the same period due to which the analysis is not sufficient to prove the direction of causality (Kazdin, 2007). Furthermore, conducting multiple assessments during the intervention was only feasible with short questionnaires which limited us in the number of items we could use. As a consequence, items measuring self-reflection were focused on youth’s reflection on their thoughts and feelings, but not on their behavior. As reflecting on behavior is part of the concept of self-reflection (Lyke, 2009), lacking an item on this aspect of self-reflection might have affected the precision of the assessment of this concept. In addition, the metacognition to meaningfully reflect on thoughts and feelings is still developing in early adolescents (Barber, 2005) and might be more difficult than reflecting on behaviors. Perhaps this is not the best developmental phase to measure self-reflection; improvements on this competence may emerge later in adolescents’ development. Last, we analyzed multiple models without adjusting the *p*-value for multiple tests. Nevertheless, our results showed a consistent pattern across the different models.

The present study makes a valuable contribution to both research and intervention development. First, it seems important to consider parallel development between constructs. Rather than examining whether self-awareness at baseline affected positive outcomes at a later time point, we showed that developments in dimensions of self-awareness, specifically emotional self-regulation and self-esteem, and positive outcomes affect each other in a parallel process meaning that changes in self-awareness are related to concurrent changes in positive youth development. Second, our study showed the importance of focusing on positive development in groups generally conceived as at risk of maladjustment. Although most studies examining prevocational students show their increased risks of developing problems (e.g., Stevens & De Looze, 2018), our study showed their positive development (regardless of the condition they participated in). Third, youth’s positive development can be affected by multiple factors among which the competencies of emotional self-regulation and self-esteem. Based on our findings and in line with CASEL’s framework, universal school-based interventions aiming to stimulate positive youth development may potentially be optimized with an additional focus on these two competencies. Examining which specific competencies are important to strengthen in intervention is eminent in order to understand how to optimize interventions and their effectiveness. This knowledge is especially important regarding universal interventions given their generally small effect sizes. To this end, the findings of the present study can be used to build on by future research by studying how emotional self-regulation and self-esteem can best be addressed in an intervention.

## Supplementary Information

Below is the link to the electronic supplementary material.

Supplementary file1 (DOC 218 KB)

## Footnotes

1 Ethnic background was coded as Western (i.e., both parents born in Europe, North-America, Oceania, Indonesia, or Japan; Central Bureau for Statistics, 2021) or non-Western (all others).
